# Supplementary material for: Genome-Wide Classification of Type I, Type II and Type III Interferon-Stimulated Genes in Chicken Fibroblasts
Source: Vaccines (Basel). 2019 Oct 25;7(4):160. doi: 10.3390/vaccines7040160 (PMC6963425; doi:10.3390/vaccines7040160)
Supplement: Supplementary file 1 [file vaccines-07-00160-s001.zip › author revision sup/Supplementary Figure 1.docx]

**Supplementary Figure**

**Genome-Wide Classification of Type I, Type II and Type III Interferon-Stimulated Genes in Chicken Fibroblasts**

Diwakar Santhakumar, Mohammed A. Rohaim and Muhammad Munir*

Division of Biomedical and Life Sciences, Faculty of Health and Medicine, Lancaster University, Lancaster, LA1 4YG, UK

* Correspondence: [muhammad.munir@lancaster.ac.uk](mailto:%20muhammad.munir@lancaster.ac.uk)


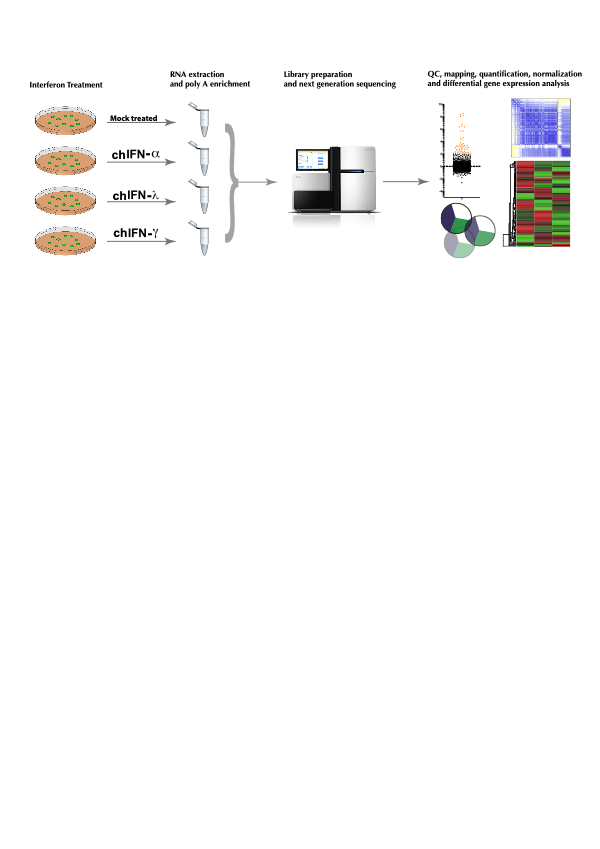


**Figure S1**. **Interferon treatment and transcriptomes workflow**. The chIL28RA DF1 cells were treated with chIFN-α, chIFN-γ, chIFN-λ or were mock treated (negative control). Total RNA was extracted followed by mRNA enrichment, mRNA fragmentation, and with polyA selection. First and second strand cDNA synthesis was conducted followed by end repair, 5’ phosphorylation, and dA tailing. Finally, adaptor ligation, PCR enrichment and sequencing were carried out followed by bioinformatics analysis, which includes QC, mapping, quantification, normalization and differential gene expression analysis.
